# Supplementary material for: Colossal barocaloric effects near room temperature in plastic crystals of neopentylglycol
Source: Nat Commun. 2019 Apr 18;10:1803. doi: 10.1038/s41467-019-09730-9 (PMC6472423; doi:10.1038/s41467-019-09730-9)
Supplement: Supplementary file 1 — Supplementary Information [file 41467_2019_9730_MOESM1_ESM.pdf]

Supplementary Information

Colossal barocaloric effects near room temperature in plastic crystals of neopentylglycol

P. Lloveras *et al.*

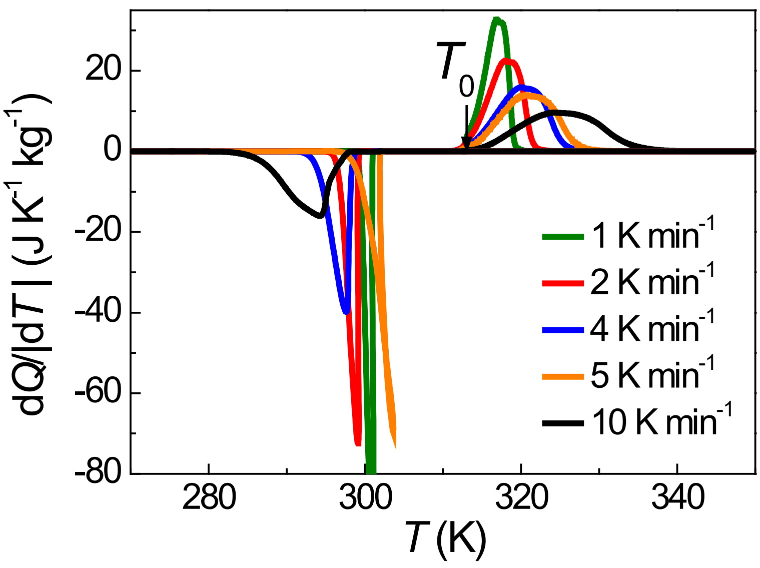

Supplementary Figure 1. Measurements of dQ/|dT| after baseline subtraction, on heating (dQ/|dT| > 0) and cooling (dQ/|dT| < 0) across the first-order PC-OC phase transition, using different temperature ramp rates. The temperature ramp rate influences the transition finish temperature on heating, and the transition start and finish temperatures on cooling. By contrast, the temperature ramp rate has nominally no effect on $|Q_0| = \left| \int_{T_1}^{T_2} \frac{\mathrm{d}Q}{\mathrm{d}T} \mathrm{d}T \right|$ across the PC-OC transition.

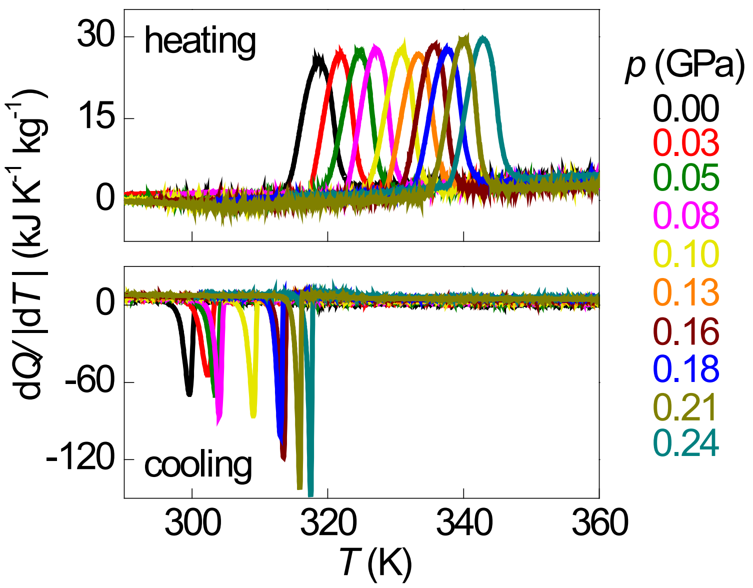

Supplementary Figure 2. Measurements of dQ/|dT| on heating (dQ/|dT| > 0) and cooling (dQ/|dT| < 0) across the first-order PC-OC transition for different values of increasing pressure p, after baseline subtraction, at ~2 K min^{-1}. Data taken using the Cu-Be Bridgman pressure cell with chromel alumel thermocouples.

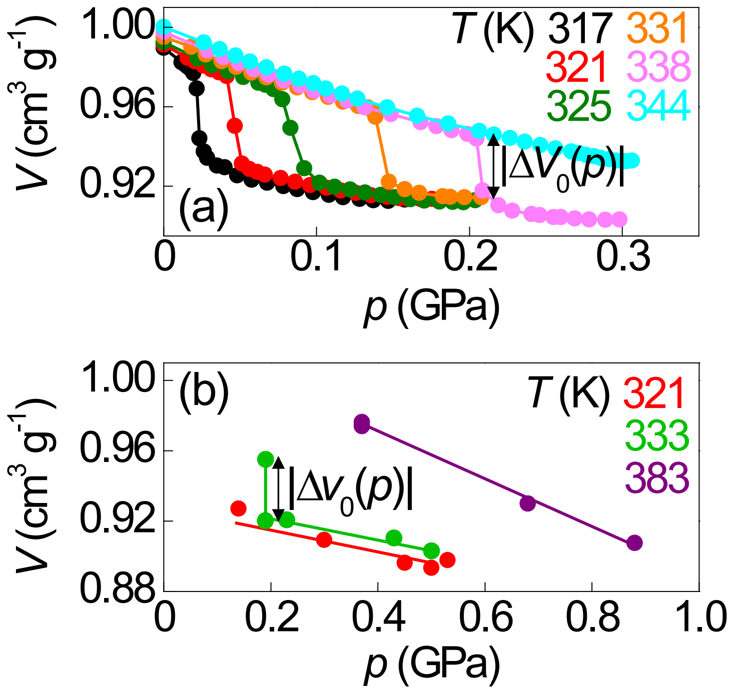

Supplementary Figure 3. (a) Pressure-dependent specific volume *V*, obtained by dilatometry while isothermally removing an applied pressure of 0.3 GPa at different measurement temperatures. (b) Pressure-dependent specific volume *V*, obtained by x-ray diffraction (XRD) while isothermally removing an applied pressure of 0.9 GPa at different measurement temperatures. Measurement errors in pressure are ±0.002 GPa for dilatometry, and ±0.05 GPa for high-pressure x-ray diffraction.

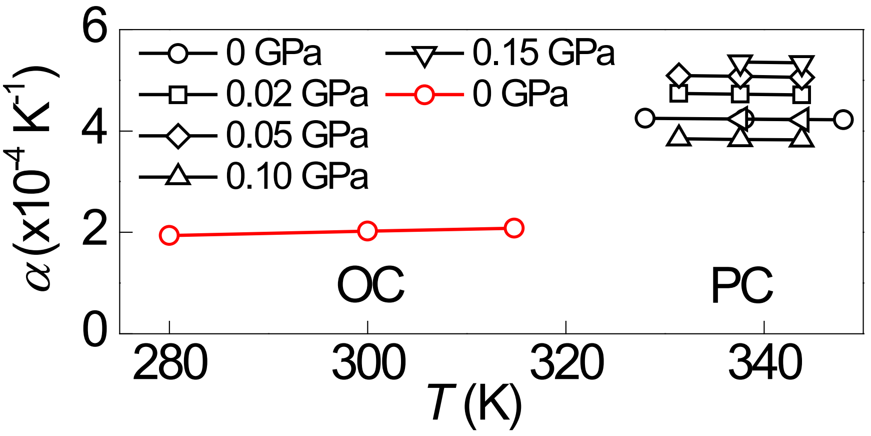

Supplementary Figure 4. Coefficient of isobaric thermal expansion α for the PC phase (black) and the OC phase (red), near the PC-OC transition, obtained from data in Figure 1(d) and Supplementary Figure 3(a). The non-monotonic changes of α with pressure for the PC phase imply a ~20% error in $(\partial V/\partial T)_p$.

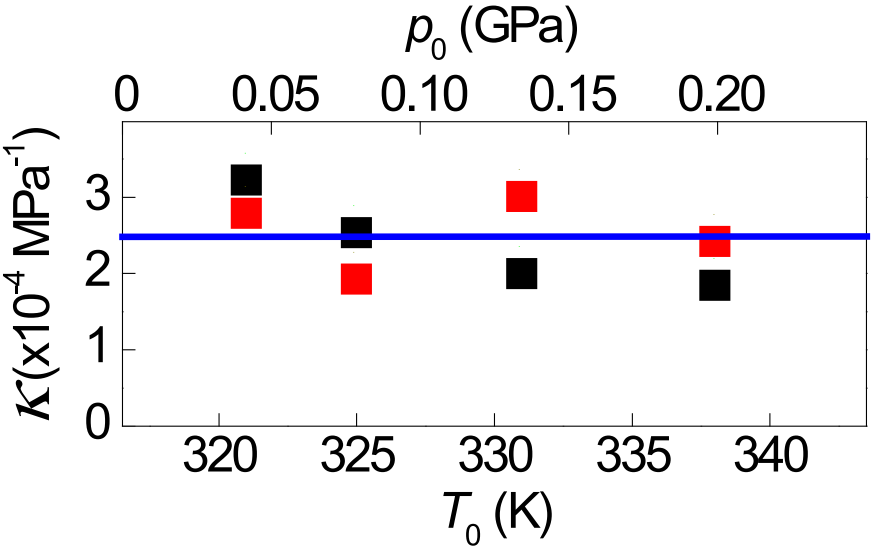

Supplementary Figure 5. Coefficient of isothermal compressibility κ for the PC phase (black symbols) and the OC phase (red symbols), near the PC-OC transition, obtained from data in Supplementary Figure 3(a). The blue line represents the average value of κ for all data.

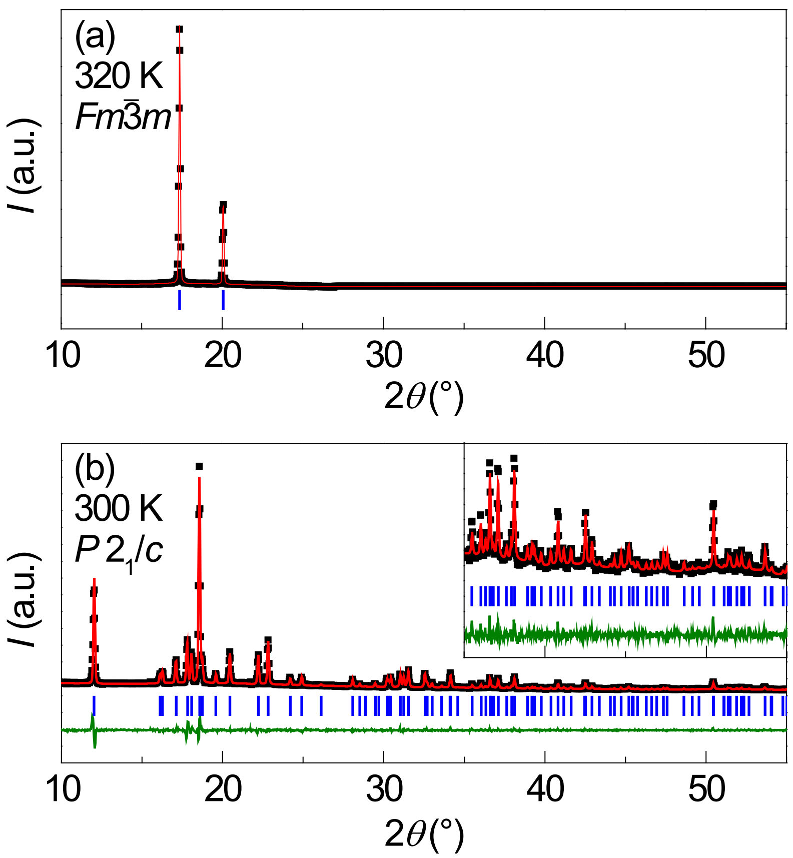

Supplementary Figure 6. Detail of selected x-ray diffraction spectra obtained (a) above and (b) below the PC-OC transition on heating, at atmospheric pressure. Black symbols are experimental data, red lines are fitted patterns, vertical blue lines indicate indexed reflections (shown overleaf).

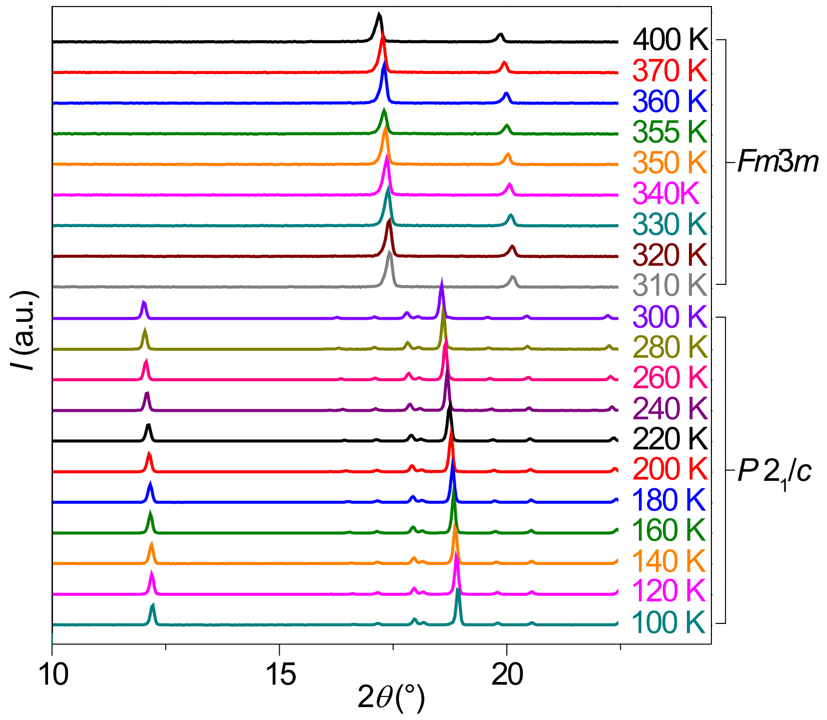

Supplementary Figure 7. Detail of all x-ray diffraction spectra obtained across the PC-OC transition on heating, at atmospheric pressure.

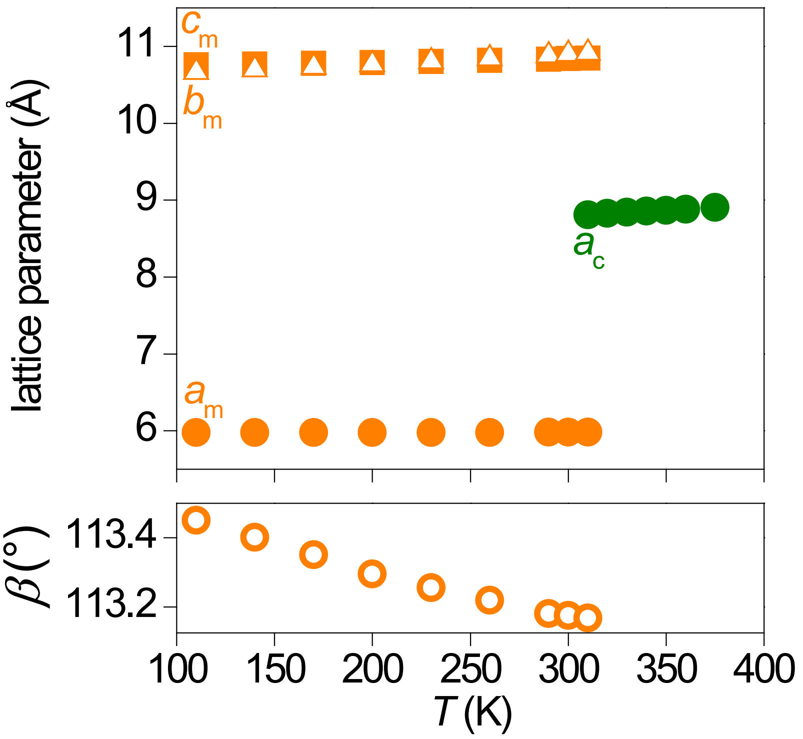

Supplementary Figure 8. Temperature dependence of lattice parameters across the PC-OC phase transition, obtained from x-ray diffraction on heating.

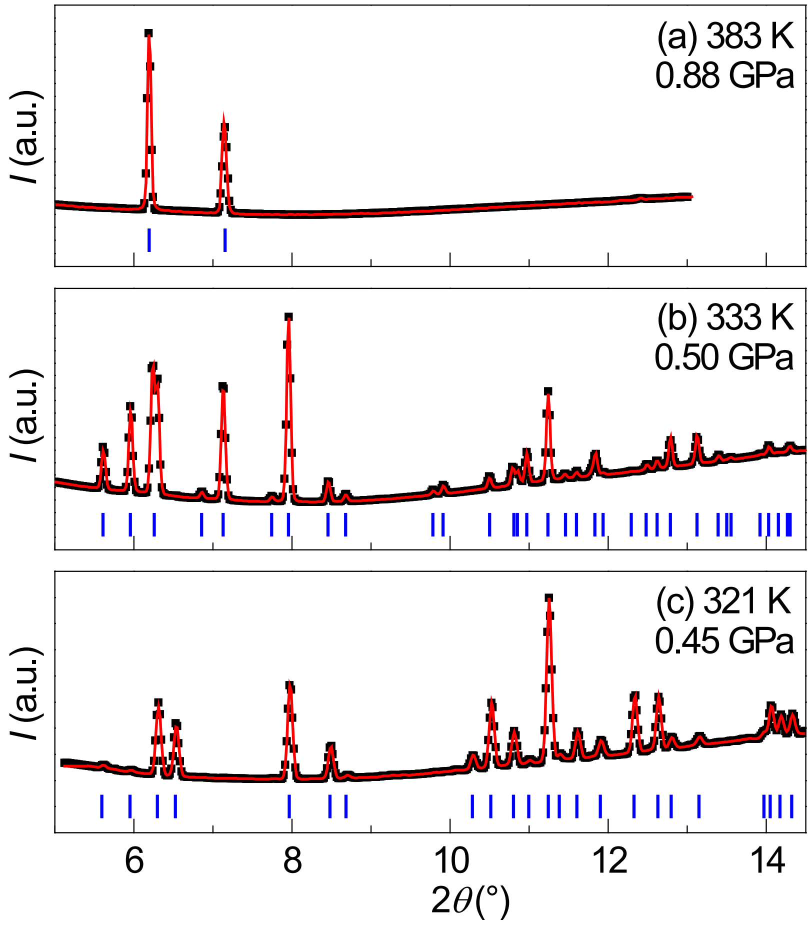

Supplementary Figure 9. Detail of selected x-ray diffraction spectra obtained under hydrostatic pressure. Black symbols are experimental data, red lines are fitted patterns, vertical blue lines indicate indexed reflections (shown overleaf).

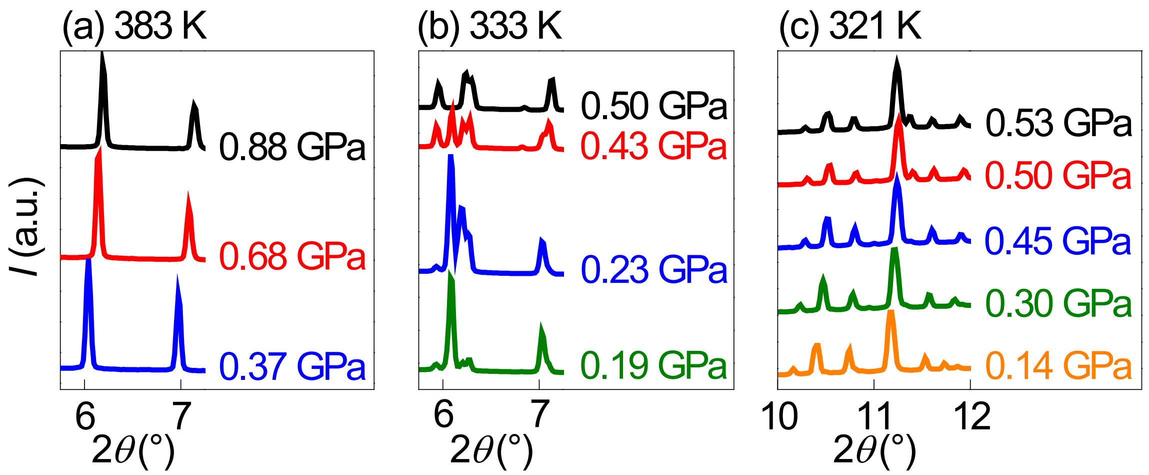

Supplementary Figure 10. Detail of x-ray diffraction spectra obtained under hydrostatic pressure, at various temperatures.

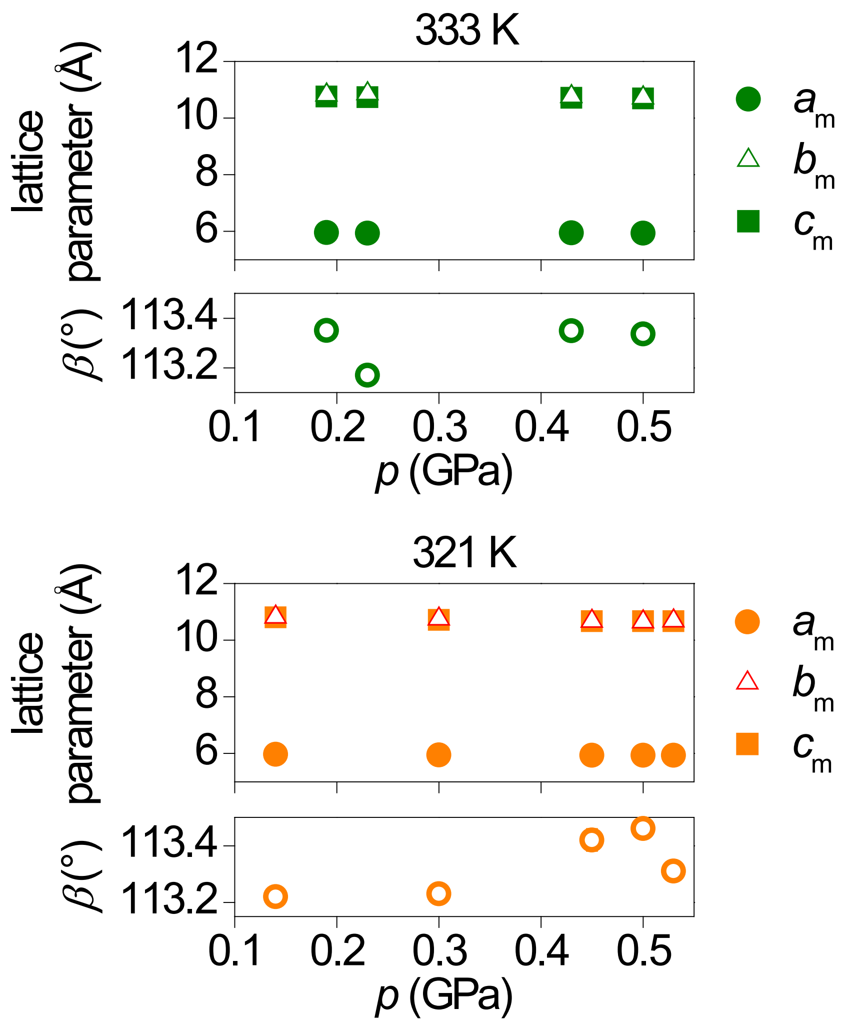

Supplementary Figure 11. Pressure dependence of lattice parameters in the OC phase, obtained from x-ray diffraction under hydrostatic pressure.

Compound	T_0 [K]	$\lvert Q_0\rvert$ [kJ kg^{-1}]	$\lvert\Delta V_0/V_0\rvert$ [%]	$\lvert dT/dp\rvert$ [K GPa^{-1}]	Suppl. Ref.
NPG	314	121	4.9	103	This work
$Ni_{49.26}Mn_{36.08}In_{14.66}$	290	7.8	0.5	18	1
$LaFe_{11.35}o_{0.47}Si_{1.2}$	250	2.8	1	94	2
$Gd_5Si_2Ge_2$	260	5.5	1	35	3
$Fe_{49}Rh_{51}$	319	3.8	1	59	4
Mn_3GaN	290	6.4	1	65	5
$(MnNiSi)_{0.62}(FeCoGe)_{0.38}$	338	21	4	75	6
$BaTiO_3$	400	0.9	0.1	55	7
$(NH_4)_2SnF_6$	110	4.3	1	157	8
$(NH_4)_2SO_4$	224	15	1	45	9
$[TPrA]Mn[dca]_3$	330	14	1.25	231	10
AgI	420	27	5	134	11

Supplementary Table 1. Phase transition properties for first-order giant barocaloric materials. For the solids in Supplementary Table 1, we show transition temperature T_0, latent heat $|Q_0|$, the relative specific volume change $|\Delta V_0|/V_0$, and the tunability of transition temperature with pressure $|dT/dp|$. For NPG, $|dT/dp|$ was obtained by averaging dT/dp on heating and cooling for $p < 0.1$ GPa [Figure 2(c)].

T = 300 K				*T* = 320 K	
**2*θ* (°)**	**hkl**	**2*θ* (°)**	**hkl**	**2*θ* (°)**	**hkl**
12.03	0 1 1	36.34	-1 4 1	17.35	1 1 1
16.12	1 0 0	36.60	2 2 0	20.06	2 0 0
16.26	0 2 0	36.65	0 3 3		
17.11	-1 1 1	36.82	1 4 0		
17.82	0 0 2	37.11	-2 2 3		
18.08	1 1 0	37.63	0 4 2		
18.57	0 2 1	38.12	2 1 1		
18.75	-1 0 2	37.94	1 3 2		
19.59	0 1 2	38.93	-2 1 4		
20.46	-1 1 2	39.20	-2 3 1		
22.23	-1 2 1	39.36	-2 3 2		
22.84	1 1 1	39.77	0 2 4		
24.20	0 2 2	40.37	1 2 3		
24.91	-1 2 2	40.83	2 2 1		
26.11	0 3 1	41.63	-2 3 3		
28.08	0 1 3	42.46	0 5 1		
28.52	1 0 2	42.54	-1 1 5		
28.87	-1 3 1	42.93	0 4 3		
29.49	1 3 0	43.39	2 0 2		
29.69	1 1 2	44.07	0 3 4		
30.23	-1 2 3	44.31	-1 5 1		
30.32	-2 0 2	44.73	1 5 0		
30.43	0 3 2	45.22	-2 1 5		
31.03	-1 3 2	45.42	0 5 2		
31.25	-2 1 1	45.49	1 0 4		
31.54	0 2 3	45.75	-2 3 4		
32.57	2 0 0	46.29	1 1 4		
32.69	1 3 1	46.63	2 2 2		
33.00	1 2 2	46.95	2 4 0		
33.60	2 1 0	47.39	-1 4 4		
34.12	0 4 1	47.60	-2 2 5		
34.15	-1 1 4	48.63	0 2 5		
34.59	-2 2 2	49.16	-1 5 3		
35.48	-1 3 3	49.57	0 4 4		
36.04	0 0 4				

Supplementary Table 2. Indexed reflections for the x-ray diffraction patterns shown in Supplementary Figure 4.

T = 321 K, *p* = 0.45 GPa		*T* = 333 K, *p* = 0.50 GPa		*T* = 383 K, *p* = 0.88 GPa	
**2*θ* (°)**	**hkl**	**2*θ* (°)**	**hkl**	**2*θ* (°)**	**hkl**
5.59	1 0 0	5.61	1 0 0	6.19	1 1 1
5.95	-1 1 1	5.96	-1 1 1	7.15	2 0 0
6.30	1 1 0	6.26	0 0 2		
6.52	0 2 1	6.86	0 1 2		
6.52	-1 0 2	7.13	-1 1 2		
7.96	1 1 1	7.74	-1 2 1		
8.48	0 2 2	7.95	1 1 1		
8.69	-1 2 2	8.46	0 2 2		
10.28	1 3 0	8.68	-1 2 2		
10.52	-1 2 3	9.78	0 1 3		
10.80	-2 1 1	9.91	1 0 2		
11.00	0 2 3	10.50	-1 2 3		
11.24	2 0 0	10.81	-2 1 1		
11.38	1 3 1	10.85	-2 1 2		
11.60	2 1 0	10.97	0 2 3		
11.90	0 4 1	11.24	2 0 0		
12.33	-1 3 3	11.46	1 2 2		
12.63	-1 4 1	11.60	2 1 0		
12.63	2 2 0	11.83	-1 1 4		
12.80	1 4 0	11.94	-2 2 2		
12.80	-2 2 3	12.29	-1 3 3		
13.15	2 1 1	12.48	0 0 4		
13.97	1 2 3	12.62	2 2 0		
14.05	2 2 1	13.92	1 2 3		
14.17	2 3 0	14.03	2 2 1		
14.32	-2 3 3	14.15	2 3 0		
		14.27	-2 2 4		
		14.30	-2 3 3		

Supplementary Table 3. Indexed reflections for the x-ray diffraction patterns shown in Supplementary Figure 7.

Supplementary References

[1] Mañosa, Ll., González-Alonso, D., Planes, A., Bonnot, E., Barrio, M., Tamarit, J.-Ll., Aksoy, S. & Acet, M. Giant solid-state barocaloric effect in the Ni–Mn–In magnetic shape-memory alloy. *Nat. Mater.* **9**, 478-481 (2010).

[2] Mañosa, Ll., González-Alonso, D., Planes, A., Barrio, M., Tamarit, J.-Ll., Titov, I. S., Acet, M., Bhattacharyya, A. & Majumdar, S. Inverse barocaloric effect in the giant magnetocaloric La– Fe – Si– Co compound. *Nat. Commun.* **2**, 595 (2011).

[3] Yuce, S., Barrio, M., Emre, B., Stern-Taulats, E., Planes, A., Tamarit, J.-Ll., Mudryk, Y., Gschneidner Jr, K. A., Pecharsky, V. K. & Mañosa, Ll. Barocaloric effect in the magnetocaloric prototype Gd5Si2Ge2. *Appl. Phys. Lett.* **101**, 071906 (2012).

[4] Stern-Taulats, E., Planes, A., Lloveras, P., Barrio, M., Tamarit, J.-Ll., Pramanick, S., Majumdar, S., Frontera, C. & Mañosa, Ll. Barocaloric and magnetocaloric effects in $Fe_{49}Rh_{51}$. *Phys. Rev. B* **89**, 214105 (2014).

[5] Matsunami, D., Fujita, A., Takenaka, K. & Kano, M. Giant barocaloric eect enhanced by the frustration of the antiferromagnetic phase in Mn3GaN. *Nat. Mater.* **14**, 73-78 (2015).

[6] Samanta, T., Lloveras, P., Saleheen, A. U., Lepkowski, D. L., Kramer, E., Dubenko, I., Adams, P. W., Young, D. P., Barrio, M., Tamarit, J. Ll., Ali, N. & Stadler, S. Barocaloric and magnetocaloric effects in $(MnNiSi)_{1-x}(FeCoGe)_x$. *Appl. Phys. Lett.* **112**, 021907 (2018).

[7] Stern-Taulats, E., Lloveras, P., Barrio, M., Defay, E., Egilmez, M., Planes, A., Tamarit, J.-Ll., Mañosa, Ll., Mathur, N. D. & Moya, X. Inverse barocaloric effects in ferroelectric BaTiO3 ceramics. *APL Mater.* **4**, 091102 (2016).

[8] Flerov, I. N., Kartasheva, A. V., Gorev, M. V., Bogdanov, E. V., Mel'nikova, S. V., Molokeev, M. S., Pogoreltsev, E. I. & Laptashf, N. M. Thermal, structural, optical, dielectric and barocaloric properties at ferroelastic phase transition in trigonal (NH4)2SnF6: A new look at the old compound. *J. Fluorine Chem.* **183**, 1-9 (2016).

[9] Lloveras, P., Stern-Taulats, E., Barrio, M., Tamarit, J.–Ll., Crossley, S., Li, W., Pomjakushin, V., Planes, A., Mañosa, Ll., Mathur N. D. & Moya, X. Giant barocaloric effect at low pressure in ferrielectric ammonium sulphate. *Nat. Commun.* **6**, 8801 (2015).

[10] Bermúdez-García, J. M., Sánchez-Andújar, M., Castro-García, S., López-Beceiro, J., Artiaga R. & Señarís-Rodríguez M. A. Giant barocaloric effect in the ferroic organic-inorganic hybrid [TPrA][Mn(dca)3] perovskite under easily accessible pressures. *Nat. Commun.* **8**, 15715 (2017).

[11] Aznar, A., Lloveras, P., Romanini, M., Barrio, M., Tamarit, J.-Ll., Cazorla, C., Errandonea, D., Mathur, N. D., Planes, A., Moya, X. & Mañosa, Ll. Giant barocaloric effects over a wide temperature range in superionic conductor AgI. *Nat. Commun.* **8**, 1851 (2017).
